# EefR mutations drive sanguinarine resistance by activating cryptic multidrug efflux pumps in AcrB-Null *Escherichia coli*

**DOI:** 10.1080/21505594.2025.2566244

**Published:** 2025-09-29

**Authors:** Hao-Jie Shen, Jun-Feng Wang, Zheng-Jie Xue, Jia-Qi Shi, Bing Chen, Dong-Min Chen, Ni-Pi Chen, Yu-Dong Li, Chao-Dong Qian

**Affiliations:** a College of Life Sciences, Zhejiang Chinese Medical University, Hangzhou, China; b School of Food sciences and Biotechnology, Zhejiang Gongshang University, Hangzhou, China

**Keywords:** *Escherichia coli*, sanguinarine, TetR family regulator, efflux pump, resistance-nodulation-cell division (RND) pump, multidrug resistance

## Abstract

Antimicrobial resistance is one of the greatest threats to global health. A thorough comprehension of the underlying processes through which bacteria evolve the ability to withstand diverse stressors is vital for the development of efficacious strategies to address this challenge. This study aimed to elucidate the mechanisms underlying the adaptation of an *acrB*-deficient strain of *Escherichia coli* ATCC 35218 (35218m) to sanguinarine (SAN), the main component of phytobiotic derived from *Macleaya cordata*. Analysis of the *in vitro*-selected SAN-resistant clones of strain 35218m led to the identification of a functionally uncharacterized TetR regulator, EefR. The regulator represses its own expression and that of four neighboring genes forming the *eefRABCD* operon, encoding components of a tripartite RND-efflux pump (EefABC) and a putative MFS-type exporter (EefD). Overexpression of these pumps reduces the susceptibility of *acrB*-deficient *E. coli* to SAN and structurally diverse antibiotics. The *eefR-eefABC-eefD* cluster, widely distributed across Enterobacteriaceae genomes, is also found on plasmids in several *E. coli* isolates, indicating a significant risk for the rapid spread of this multidrug resistance mechanism.

## Introduction

The crisis regarding antibiotic resistance represents a significant threat to global public health security in the 21st century [1,2]. According to the most recent statistical data, approximately 1.27 million deaths worldwide are directly attributable to drug-resistant bacterial infections each year, and the mortality associated with these infections is projected to exceed that caused by cancer by 2050 [3]. This critical situation is closely associated with the excessive deployment of antibiotics in the medical, livestock, and agricultural sectors [4]. Of particular concern is the protracted antibiotic utilization in animal husbandry, which has been recognized as a pivotal driver for the emergence and horizontal dissemination of antimicrobial resistance genes. This practice has prompted global regulatory bans on antibiotic growth promoters (AGPs), with numerous countries restricting their use in livestock production since 2006 [5,6]. In this context, plant-derived phytochemicals have garnered increasing attention in livestock production as potential alternatives to AGPs [6,7].

**Figure 1.**
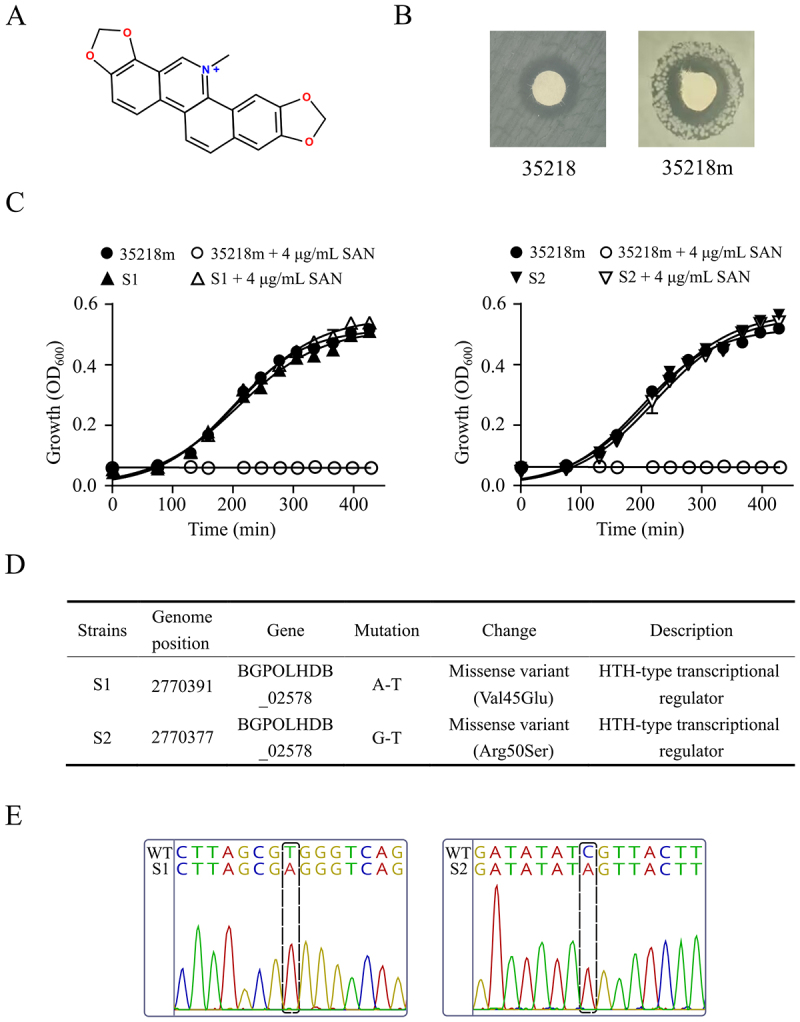
Sanguinarine-resistant mutants of *acrB*-deficient *E. coli* 35218m. A, the structure of sanguinarine. B, resistant strains emerged within the inhibition zone of the sanguinarine disc. C, growth curves of the 35218m strain and its mutant strains in the presence or absence of 4 μg/mL sanguinarine. D, single nucleotide polymorphisms (SNP) in mutant stains of 35218m compared to the parent strain. E, sequencing analysis of the mutation sites, with black dashed boxes highlighting the locations of the mutations.

Extracts of *Macleaya cordata* and its principal bioactive alkaloid, sanguinarine (SAN, Figure 1(A)), have been used extensively in livestock production due to their broad-spectrum antimicrobial properties against zoonotic pathogens. A recent study showed that a single exposure to a SAN-containing phytobiotic did not result in the spread of antibiotic resistance genes [8]. However, the emergence of multidrug-resistant mutants of *Escherichia coli* has been documented in cases of repeated exposure to subinhibitory SAN [9]. Whole-genome sequencing identified loss-of-function mutations in TetR-family transcriptional regulators (*marR*/*acrR*) as the primary molecular determinant, which drives overexpression of the AcrAB-TolC efflux complex via derepression of this RND-type transporter system. The disruption of the gene *acrB* resulted in a 16-fold decrease in the minimum inhibitory concentration (MIC) of SAN against *E. coli* strains [9]. This discovery elucidates the dual role of AcrAB-TolC in mediating both intrinsic and adaptive resistance to SAN and highlights the therapeutic potential of combination therapies that incorporate efflux pump inhibitors.

Despite the heightened sensitivity exhibited by *acrB*-deficient *E. coli* mutants to SAN in comparison to wild-type strains, these mutants demonstrate an elevated propensity to elicit resistant revertant mutations. This tendency not only promotes the generation and spread of resistance genes, but also affects the efficacy of combination therapies with efflux pump inhibitors. To obtain more information about the effect of SAN on bacterial resistance 35218m, a mutant of *E. coli* ATCC 35218 with a deficient *acrB* [9] was selected for the experiment. Stable SAN-resistant mutants were isolated by single-step selection and their cross-resistance to different antibiotics was determined. The mechanisms of SAN-induced antibiotic resistance were investigated in detail using a combination of whole genome sequencing, gene knockout, quantitative real-time PCR (qRT-PCR) and transcriptomics analysis. The primary cause of this resistance was identified as a mutation in a functionally uncharacterized gene, BGPOLHDB_02578, which was found to encode a TetR family regulator EefR. EefR repressed the expression of its own and four neighboring efflux pump genes in the *E. coli* strain. The results would further contribute to our understanding of the dissemination of antibiotic resistance induced by the phytochemical.

## Materials and methods

### Bacterial strains, plasmids, and chemical reagents

The bacterial strains and plasmids used in this study were described in Table S1. Most bacterial strains used here were derived from 35218m, a mutant of *E. coli* ATCC 35218 with a deficient *acrB* [9]. Luria-Bertani (LB) (NaCl, 5.0 g/L; yeast extract, 5.0 g/L; and tryptone, 10.0 g/L; pH 7.0) was a commonly utilized medium for the routine cultivation of bacteria, whereas Mueller-Hinton (MH) medium (Oxoid, UK) was employed for the assessment of drug susceptibility. Unless specified, all cultures were incubated at 37°C from 18 to 24 hours. When needed, appropriate antibiotics for plasmid selection and maintenance were used at the following concentrations: ampicillin at 100 μg/mL, tetracycline at 20 μg/mL, and kanamycin at 50 μg/mL. SAN ( > 98%) was purchased from Shanghai Macklin Biochemical Co., Ltd (China). Other chemicals and antibiotics were purchased from Sigma-Aldrich (St. Louis, MO, United States) or Aladdin Bio-Chem Technology Co., Ltd (Shanghai, China).

### Drug susceptibility test

The MIC was employed to determine the antimicrobial susceptibility of microorganisms. The MIC was determined by using the broth microdilution method according to CLSI 2018 guidelines [10]. Briefly, the antimicrobial agent was subjected to serial twofold dilutions in a 96-well microplate containing MH broth. An equal volume of a bacterial suspension, standardized to a concentration of ~ 1.0 × 10^6^ CFU/mL, was then added to each well containing the diluted compounds. The microplates were then incubated at 37°C for 18–20 hours under aerobic conditions. The MIC was defined as the lowest concentration of an antimicrobial agent that prevented the visible growth of a microorganism under tested conditions.

### Mutants selection and whole genome sequencing

A one-step selection procedure was used to isolate clones resistant to SAN. Briefly, a 100 μL inoculum of strain 35218m containing approximately 3 × 10^10^ CFU/mL of the strain was spread on LB plates supplemented with varying concentrations of SAN (4 to 64 μg/mL). All plates were incubated at 37°C for 48–96 hours. After incubation, individual colonies were randomly selected, purified by streaking on drug-free LB plates and their susceptibility to SAN was measured by MIC assay. Two colonies (designated S1 and S2) were selected for further investigation on a random basis. Genomic DNA was extracted from the SAN-resistant mutant strains and their parental strain using the ZR Fungal/Bacterial Genomic DNA ExtractionKit (Zymo Research Corp, Orange, CA, United States) according to the manufacturer’s protocols. To determine the location of the mutations, whole genome sequence analysis was performed as described in the reference [9].

### Gene knockout and complementation

The *eefR*-deficient 35218m (Δ*eefR*) was constructed by using the CRIPSR-Cas system [11]. Due to resistance issues, the original spectinomycin resistance in plasmid pTargetF was replaced with tetracycline resistance. The sgRNA sequence (GCTGGCAATTTGCGACATACTGG) was designed using the website (http://crispor.gi.ucsc.edu/) to target the NGG protospacer adjacent motif (PAM) site of the *eefR* gene. The pTargetF (*tetR*) plasmid containing a new N20 sequence (GCTGGCAATTTGCGACATAC) was constructed using the primers pTargetF-F and pTargetF-R. The single-stranded DNA repair oligonucleotide (Integrated DNA Technologies) was designed to include an *AseI* restriction site and three tandem stop codons [12]. The plasmid pTargetF-*eefR* and ssDNA fragment were introduced into 35218m-pCas competent cells via electroporation. Recombinant colonies were then selected on dual antibiotic plates containing kanamycin and tetracycline.

For functional complementation of Δ*eefR*, the *eefR* gene was amplified from chromosomal DNA of 35218m using primers pBR-*eefR*-F/pBR-*eefR*-R, and inserted it into the plasmid pBR322 (amplified by primers pBR-f/pBR-r) using One Step Seamless Cloning kit (Accurate Biology, Hunan, China). After confirmed by PCR and DNA sequencing, the recombinant plasmid pBR*eefR* were transformed into Δ*eefR* to generate strains CΔ*eefR*. The same method was employed to introduce the wild-type *eefR* gene into the S1 and S2 strains, thereby yielding S1-C*eefR* and S2-C*eefR*, respectively. The primers used in this study were listed in Table S2.

### Measurement of gene expression

Total RNA was extracted from the logarithmic growth phase (OD_600_ of 0.5) of 35218m and its SAN-resistant mutants, using TRIzol® Reagent (Invitrogen, Carlsbad, CA, USA). The Nanodrop 2000 (Thermo Fisher Scientific, USA) was utilized in order to ascertain the concentration and purity of the extracted RNA. Meanwhile, agarose gel electrophoresis was employed to verify the integrity of the RNA. For the purpose of RNA sequencing, the Ribo-Zero magnetic kit (Epicenter Biotechnologies, Madison, WI, United States) was used to remove ribosomal RNA from total RNA. Library construction and HiSeq4000 (Illumina, Inc., USA)-based RNA sequencing were carried out by Shanghai Lingen Biotechnology Co., Ltd. For qRT-PCR, the process of synthesis cDNA was conducted using the Evo M-MLV RT Mix Kit (Accurate Biology, Hunan, China). The qRT-PCR was performed using the SYBR Green Premix Pro Taq HS qRT-PCR Kit (Accurate Biology, Hunan, China) on a qTOWER3G PCR system (Analytik Jena AG, Germany). The expression level of each targeted gene was normalized to that of the 16S rRNA gene. The qRT-PCR primers used in this study were listed in Table S3.

### Protein expression and purification

The *eefR* coding region was amplified by PCR from 35218m and cloned into a pET28a (+) expression vector using seamless cloning technology, resulting in a C-terminally His-tagged construct (pET28a-*eefR*). The heterologous overexpression of the recombinant protein was performed in *E. coli* BL21 (DE3). An overnight culture of BL21 (DE3) was inoculated at 1% into fresh LB medium and incubated at 37°C with shaking at 150 rpm for 4 hours. Isopropyl β-D-1-thiogalactopyranoside (IPTG) was then added to a final concentration of 1 mM, and the culture was further incubated at 25°C for 12 hours to induce protein expression. Subsequently, the culture was pelleted by centrifugation, resuspended in a Tris buffer (50 mM Tris-HCl, 500 mM NaCl, 5% glycerol, pH 8) and lysed by sonication on ice. Thereafter, the supernatant obtained by centrifugation was added to Ni-NTA resin (Ni Bestarose FF, Jiaxing, Zhejiang, China) pre-equilibrated with Tris buffer. The resin was then washed with Tris buffer containing 10–60 mM imidazole, followed by elution with Tris buffer containing 120 mM imidazole. The purified protein was then subjected to an assessment of its purity via SDS-PAGE, after which it was confirmed to be the target protein by western blot analysis using an anti-His tag antibody (HA1006, Hangzhou, China).

### Electrophoretic mobility shift assay

The non-radioactive electrophoretic mobility shift assay (EMSA) was performed using biotin-labeled DNA fragments of 129 base pairs in length and located upstream of the *eefR* promoter region. To obtain biotinylated and non-biotinylated probes, PCR amplification was performed using the primers listed in Table S2. The EMSA reaction mixture containing the DNA probes with varying molar concentrations of EefR protein was incubated in binding buffer at 25°C for 20 minutes, followed by electrophoretic separation on a 6% native-PAGE gel at 4°C. After electrophoresis, the gel was transferred to a positively charged nylon membrane in accordance with the EMSA kit protocol (Beyotime, Shanghai, China). The results were then subjected to visualization using the ChemiDoc Imaging System.

### Measurement of green fluorescent protein expression

The pBR322-based GFP reporter plasmids were constructed by Gibson assembly [13]. The *gfp* gene was amplified from pGFPuv plasmid using the primer pair *gfp*-F and *gfp*-R. The Gibson assembly fragments of pBR322 and the upstream promoter region of the *eefR* (*P*_*eefR*_) were amplified with the primer pairs pBR-GFP-F/R and *P*_*eefR*_-F/R, respectively. The resulting PCR products were then assembled, resulting in the plasmid pBR-*P*_*eefR*_-GFP. Consequently, the construction of pBR-*P*_*eefR*_-GFP-EefR was achieved by amplifying the *eefR* gene from strain 35218m using the primers *eefR*-F2/R2, followed by its insertion into the pBR-*P*_*eefR*_-GFP plasmid. This resulted in the formation of the plasmid pBR-*P*_*eefR*_-GFP-EefR, which carries the *P*_*eefR*_ promoter to drive the expression of the GFP and the tetracycline promoter to drive the expression of the *eefR* gene. Plasmids pBR-*P*_*eefR*_-GFP-EefR^S1^ and pBR-*P*_*eefR*_-GFP-EefR^S2^ were constructed using primers S1-F/R and S2-F/R based on plasmid pBR-*P*_*eefR*_-GFP-EefR. In these constructs, the genes coding for EefR^S1^ and EefR^S2^ were sourced from the mutant strains S1 and S2 of the 35218m strain, respectively. These expression plasmids were transformed into *E. coli* DH5α, 35218m, or Δ*eefR*.

To quantify GFP expression, bacterial cells were grown to log phase, washed with PBS buffer and re-suspended to an OD_600_ of approximately 1. GFP fluorescence was then analyzed using a SpectraMax i3x Multi-Mode Microplate Reader (Molecular Devices, Sunnyvale, CA, USA) with an excitation wavelength of 395 nm and an emission wavelength of 509 nm.

### Cloning of the efflux pump genes

pBR322-based plasmids were constructed using seamless cloning to incorporate target gene sequences into vector segments; both fragments had approximately 20 base pairs of homologous sequences at their ends. Primer pairs pBR-F1/pBR-R1, pBR-F1/pBR-R2, pBR-F1/pBR-R3, and pBR-F2/pBR-R4 were utilized to amplify the vector segments of pBR*eefAB*, pBR*eefABC*, pBR*eefABCD*, and pBR*eefD*, respectively. The primer pairs *eefA*-F/*eefB*-R, *eefA*-F/*eefC*-R, *eefA*-F/*eefD*-R1, and *eefD*-F/*eefD*-R were then used to amplify the target gene sequences of the above four plasmids. All target genes were inserted downstream of the tetracycline resistance gene promoter in the pBR322 plasmid backbone. The four plasmids were introduced into *E. coli* DH5α Δ*acrB* through heat shock transformation, followed by further experimental procedures. The constructs were confirmed using PCR and Sanger sequencing.

### Distribution analysis of bacteria harbouring the *eefR-eefABC-eefD* gene cluster

The nucleic acid sequence of the *eefRABCD* gene cluster from *E. coli* ATCC 35218 was subjected to alignment and search using the BLAST tool on the NCBI website, with screening conditions set to coverage ≥ 99% and sequence identity ≥ 90%. A total of 2,976 bacterial strains with complete genomes were obtained from the screening results for subsequent analysis. The bacterial strains identified from the dataset were subjected to source analysis, which categorized them as human-associated, animal-associated, or environment-associated. Phylogenetic typing was conducted on the strains of *E. coli* using the ClermonTyping tool (http://clermontyping.iame-research.center). The sequence information of the gene cluster was listed in Table S5. The data were downloaded and curated from the NCBI database on 3 March 2025.

### Statistical analyses

All experiments were performed in biological triplicates and data were presented as means ± SD. Statistical analysis was carried out using Graphpad Prism 10 (Graph Pad Software, USA). T test was used to calculate *p* values and significant differences (*, *p* < 0.05; **, *p* < 0.01; ***, *p* < 0.001; and ****, *p* < 0.0001).

## Results

### The *acrB*-deficient 35218m tends to develop multidrug resistance after treatment with SAN

SAN had been reported to be a good substrate recognized by AcrB, the key substrate-binding subunit of the *E. coli* AcrAB-TolC tripartite efflux pump [9]. The *acrB*-deficient mutant of *E. coli* ATCC 35218, designated 35218m, showed high susceptibility to SAN with a 16-fold reduced MIC compared to its parental strain. Interestingly, growing colonies of 35218m were often observed within the zone of inhibition around the SAN disc in susceptibility testing (Figure 1(B)). To gain more information about the effect of SAN on bacterial resistance, antibiotic-resistant mutants of 35218m were isolated by single-step selection on plates containing different concentrations of SAN. Generation of resistant mutants on plates containing 32 µg/mL of SAN was achieved at a mutation rate of approximately 0.5 × 10^−7^. Two colonies (S1 and S2) were randomly isolated for further investigation. The MIC of 35218m was 1 µg/mL of SAN, while all mutants showed an increase in resistance with MICs of 8 µg/mL of this phytochemical (Table 1). Both mutants showed no significant difference in growth behavior compared to the parental strain 35218m in drug-free LB (Figure 1(C)), and their resistance to SAN remained stable over three passages on drug-free agar. The susceptibility of the mutant strains to a series of standard antibiotics was also determined, revealing that the MICs for erythromycin, chloramphenicol, fusidic acid, clindamycin and dequalinium chloride increased ≥ 4-fold compared to the parental strain, whereas no such increase was observed for kanamycin and polymyxin B (Table 1). Notably, single-step selection of wild-type *E. coli* ATCC 35218 with functional AcrB has proven difficult to generate stable drug-resistant mutants [9].

**Table 1.** Mics of sanguinarine and other antimicrobial agents against 35218m and its derivative strains.

Compound	MIC (μg/mL)						
	35218m	S1	S2	Δ*eefR*	CΔ*eefR*	S1-C*eefR*	S2-C*eefR*
Sanguinarine	1	8	8	8	0.5	4	4
Erythromycin	4	64	64	64	2	16	32
Clindamycin	8	32	32	32	4	16	16
Fusidic acid	8	64	64	64	8	32	32
Chloramphenicol	64	256	256	256	32	64	64
Dequalinium chloride	4	32	32	32	4	8	8
Kanamycin	2	2	2	2	2	2	2
Polymyxin B	2	2	2	2	2	2	2

### Mutation in *eefR* is responsible for the multidrug resistance of the mutant strains

The complete genomes of the two mutant strains were subjected to whole-genome sequencing. Compared with the parental strain, both mutants exhibited missense mutations in a shared gene, BGPOLHDB_02578 (*eefR*), which was annotated as encoding a helix-turn-helix domain-containing protein (Figure 1(D), Table S5). Specifically, S1 harbored a C to T transition at genome position 2,770,391 within *eefR*, causing an amino acid change (Val45Glu), whereas a G to T transition at genome position 2,770,377 (Arg50Ser) was presented in mutant strain S2. Subsequent confirmation of each variation was achieved through PCR amplification and sequencing (Figure 1(E)). Five additional SAN-resistant isolates were subsequently picked from plates containing 32 µg/mL of SAN, and the *eefR* genes of these mutants were all found to contain missense and/or frameshift mutations (Table S4).

In order to verify the involvement of *eefR* in resistance to SAN, a deletion mutant of 35218m (Δ*eefR*) was created, in conjunction with its complemented strain (CΔ*eefR*). MICs of these strains were then determined. As demonstrated in Table 1, the deletion of *eefR* resulted in a significant increase in the resistance of 35218m to SAN, with an MIC that was comparable to that observed in the resistant mutants. Conversely, introduction of WT *eefR* into Δ*eefR* fully restored the susceptibility to SAN. The changes in the susceptibility of several other antibacterials to *eefR*-deficient or complemented strains were similar to those observed for SAN. These results suggested that the loss of the normal function of EefR may be responsible for the multidrug resistance (MDR) of the isolated mutant strains. Interestingly, when WT *eefR* was introduced into either S1 or S2, the susceptibility to the phytochemical and other antibiotics was only partially restored. One potential explanation for this phenomenon was that the *eefR* missense mutants identified in strains S1 and S2 acted as dominant-negative mutations, thereby interfering with the function of the wild-type EefR protein.

### EefR is an uncharacterized TetR regulator and regulates its own expression

**Figure 2.**
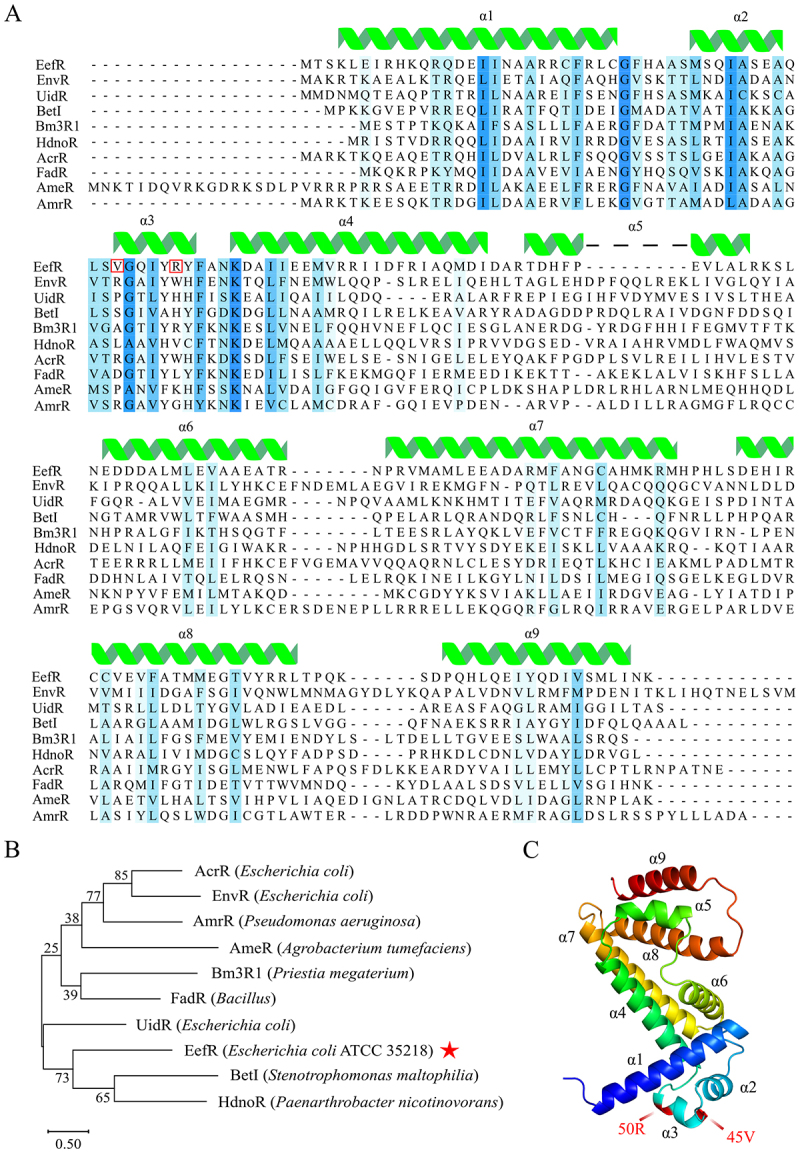
EefR characterized as a TetR-family regulator. A, multiple sequence alignment of EefR and its homologs. The secondary structure annotations above the aligned sequences correspond to the EefR protein. The transparent blue boxes indicate sequence conservation, with darker shades representing greater similarity. The red box highlights the mutation sites S1 and S2 on EefR. B, the phylogenetic tree was constructed according to the sequence alignment of EefR and its homologs. C, the structure of EefR obtained with AlphaFold3. The mutation sites of S1 and S2 are highlighted in red font.

EefR contained 188 amino acids with a theoretical molecular weight of 21.79 KDa. According to SMART analysis [14], EefR was predicted to have a possible DNA-binding HTH motif encompassing amino acids 16–62 at the N-terminus and a putative ligand-binding domain at the C-terminus. The BLAST search results in NCBI server [15] showed that EefR was highly similar (amino acid identity of > 99%, coverage 100%) to a number of proteins annotated as transcriptional regulators of the TetR/AcrR family in many *E. coli* strains. However, among the sequences with the highest identity, none had undergone functional characterization, making EefR the first to be functionally characterized in this work. Alignment performed with PRALINE server [16] employing sequences of characterized TetR regulators showed that EefR had the highest sequence identity with *E. coli* UidR/GusR (21%) [17,18], and shared more conserved amino acid residues in the DNA-binding domain than in the ligand-binding domain (Figure 2(A)). Despite the phylogenetic analysis indicating that EefR was a distinct entity, forming a discrete branch within the phylogenetic tree (Figure 2(B)), the model structure obtained with AlphaFold3 [19] suggested that EefR was composed of nine α-helices (Figure 2(C)), an important characteristic of most members of the TetR family [20].

**Figure 3.**
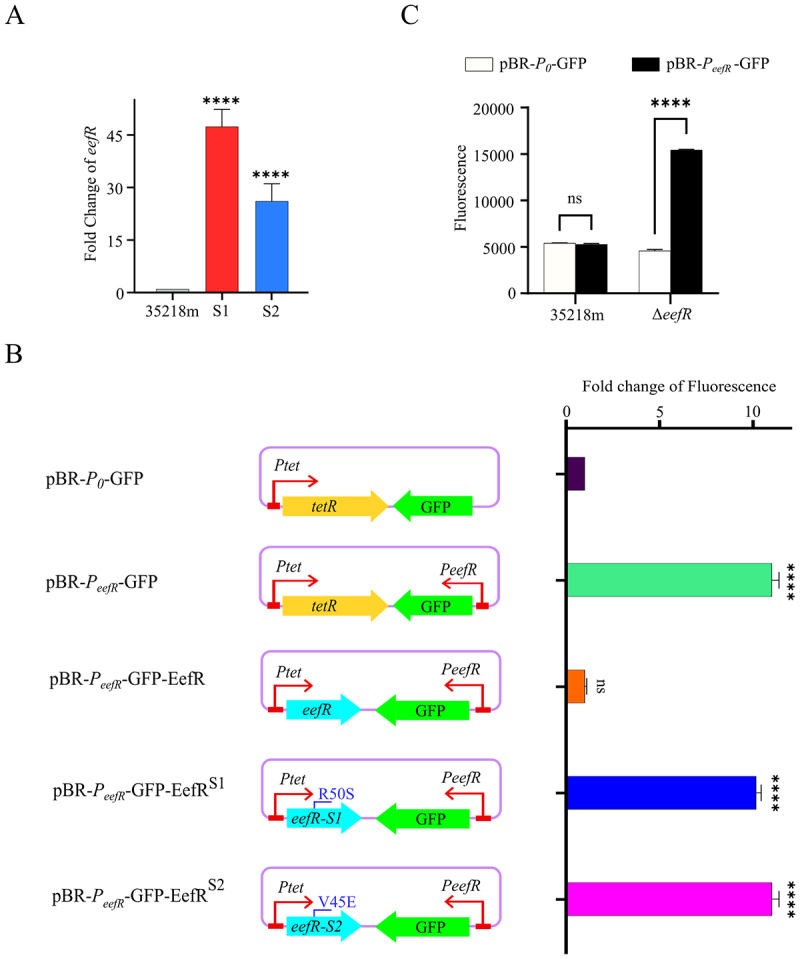
EefR acting as a self-repression regulator. A, expression levels of *eefR* in 35218m, S1 and S2. B, schematic diagrams of the GFP reporter plasmids. Plasmid pBR-*P*_*0*_-GFP, GFP expression in the absence of a promoter; pBR-*P*_*eefR*_-GFP, GFP expression under the *P*_*eefR*_ promotor (promoter of *eefR* gene); pBR-*P*_*eefR*_-GFP-EefR, GFP expression under the *P*_*eefR*_ promotor and EefR expression under the *P*_*tet*_ promotor (promoter of tetracycline resistance gene); pBR-*P*_*eefR*_-GFP^S1^, GFP expression under the *P*_*eefR*_ promotor and EefR^S1^ (EefR protein gene of S1) expression under the *P*_*tet*_ promotor; pBR-*P*_*eefR*_-GFP^S2^, GFP expression under the *P*_*eefR*_ promotor and GFP^S2^ (EefR protein gene of S2) expression under the *P*_*tet*_ promotor. On the right, the Fold change in GFP fluorescence from *E. coli* DH5α colonies harboring each of the five plasmids is illustrated. C, quantitative measurement of GFP expression in 35218m and Δ*eefR* harboring pBR-*P*_*0*_-GFP and pBR-*P*_*eefR*_-GFP.

The TetR regulator has been demonstrated to exhibit autoregulatory properties [21]. In comparison with the parental strain 35218m, a significant upregulation of the *eefR* levels in the mutant strains S1 and S2 was observed by qRT-PCR analysis (Figure 3(A)). In order to further test the hypothesis that EefR controls its own expression, a promoter-reporter construct for *eefR* (pBR-*P*_*eefR*_) was created by cloning approximately 316 bp upstream of *eefR* ahead of a *gfp* reporter gene in a promoter-less plasmid pBR322 (Figure 3(B)). Additionally, pBR-*P*_*0*_ was employed as a negative control, which lacks a promoter in front of *gfp*. Following the fusion of the *eefR* promoter to *gfp*, expression was observed in *E. coli* DH5α, with a fluorescence intensity approximately 10-fold higher than that observed in the negative control (Figure 3(B)). As anticipated, the subsequent introduction of WT *eefR* resulted in the repression of *gfp* expression, as evidenced by the lack of a significant difference in fluorescence intensity between these cells and the negative control cells (Figure 3(B)). In contrast, the introduction of the Val45Glu or Arg50Ser mutant of *eefR* from S1, or S2 had no repressive effect on *gfp* expression in *E. coli* DH5α containing the plasmid pBR-*P*_*eefR*_ (Figure 3(B)). Notably, Val45 and Arg50 were located in the predicted EefR α3-helix region (Figure 2(C)), which has been identified as an important element for TetR regulators to interact with DNA [22]. These results indicated that EefR represses the expression from its own promoter, thereby demonstrating negative auto-regulation. In agreement with this conclusion, the GFP fluorescence values were markedly elevated in the Δ*eefR* when transformed with the plasmid pBR-*P*_*eefR*_ compared to pBR-*P*_*0*_, whereas no notable alteration was discerned in the parental strain 35218m (Figure 3(C)).

### EefR binds to a palindromic sequence upstream of *eefR*

**Figure 4.**
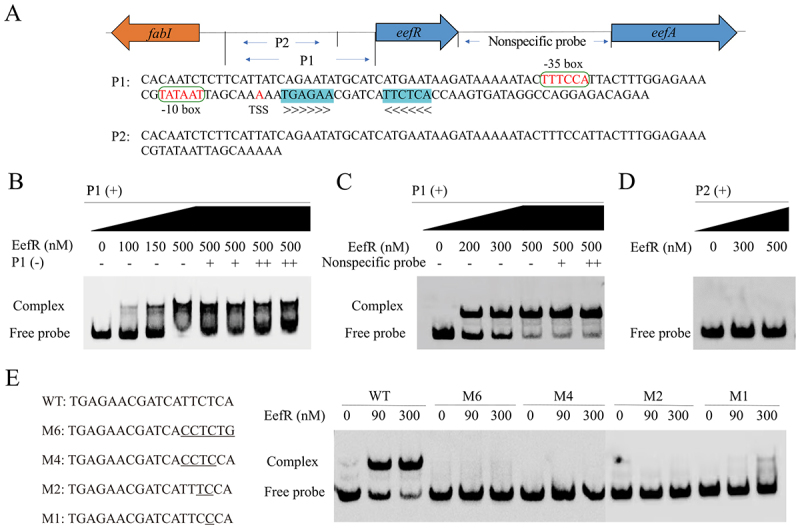
EefR binding to upstream palindromic sequence. A, P1 is a 129 base pair DNA sequence located between the genes *fabI* and *eefR*. This region comprises inverted repeat sequences, which are demarcated in green, with arrows indicating the direction of the sequence. The bases, which are highlighted in red font, represent the predicted − 35 box, −10 box and transcription start site. P2 is a DNA probe that is devoid of inverted repeat sequences. B, P1 at different concentrations binds to EefR protein, with cold probes (biotin-free probes) used for competitive inhibition; two replicates are set for each cold probe concentration. C, P1 at different concentrations binds to EefR protein, with nonspecific probes used for competitive inhibition. D, P2 at different concentrations binds to EefR protein. E, the mutant fragment M6-M1 was introduced by PCR, as shown below the wild-type (WT), with the mutated bases underlined. The mutated DNA probe was analyzed using EMSA with EefR. “+” and “-” indicate the presence or absence of the respective probe. “+” indicates that the probe concentration is the same as the fluorescent probe, while “++” indicates that the probe concentration is twice that of the fluorescent probe.

The TetR regulator is known to bind to the palindromic sequence located in the upstream region of the gene that it regulates [23]. An analysis of the upstream region of *eefR* revealed the presence of a palindrome sequence (TGAGAACGATCATTCTCA) situated upstream of the gene (Figure 4(A)). To confirm the binding of EefR directly to this region, an EMSA was conducted with upstream DNA segment of *eefR* and purified EefR protein. The *eefR* gene was cloned and overexpressed under the control of the T7 promoter in *E. coli* BL21(DE3), and the C-terminal His-tagged protein was purified using Ni-NTA resin (Figure S1). The recombinant protein was mixed with a 129 bp DNA probe (P1) derived from the upstream region of the *eefR* gene containing the palindromic sequence. As shown in Figure 4(B), increasing amounts of the purified EefR shifted the migration of the DNA probe, and a DNA-protein complex was observed. The binding was found to be highly specific, as the formation of the DNA-protein complex was impeded by the addition of increasing concentrations of the corresponding unlabeled P1 (cold probe), but not by the nonspecific probe (a 148 bp DNA fragment downstream region of the *eefR* gene) (Figure 4(C)). Furthermore, the absence of a DNA-protein complex was observed (Figure 4(D)) in the case of the P1 lacking the palindrome sequence (P2), which served to illustrate the significance of the specific sequence for recognition by EefR.

The role of the palindromic motif in mediating specific binding of EefR was further elucidated by the generation of a series of DNA probes comprising single or multiple point mutations within the palindrome sequence. The nucleotide sequence of one side that is essential for the formation of a palindrome was substituted by switching the nucleotide A/T to G/C (or vice versa). In contrast to the wild-type DNA probe, the mutations, including the one with a single base substitution, did not exhibit a significant shift in the EMSA, even at the highest protein concentration (Figure 4(E)). The findings further corroborated the hypothesis that the EefR protein exhibited a high degree of specificity in its recognition of and binding to the inverted repeat present within the *eefR* promoter region.

Subsequently, an additional search was conducted for potential EefR targets within the *E. coli* ATCC 35218 genome. The 18-bp palindrome sequence (TGAGAANNNNNNTTCTCA) was employed as the input for scanning the accessible upstream regions of *E. coli* genes with the MAST tool from the MEME Suite [24]. A search of the upstream regions of other genes revealed no evidence of a similar repeat, which suggested that the regulation of EefR was directly mediated by binding EefR only to its own promoter region.

### EefR regulates the expression of four neighboring efflux pump genes that form an operon with *eefR*

**Figure 5.**
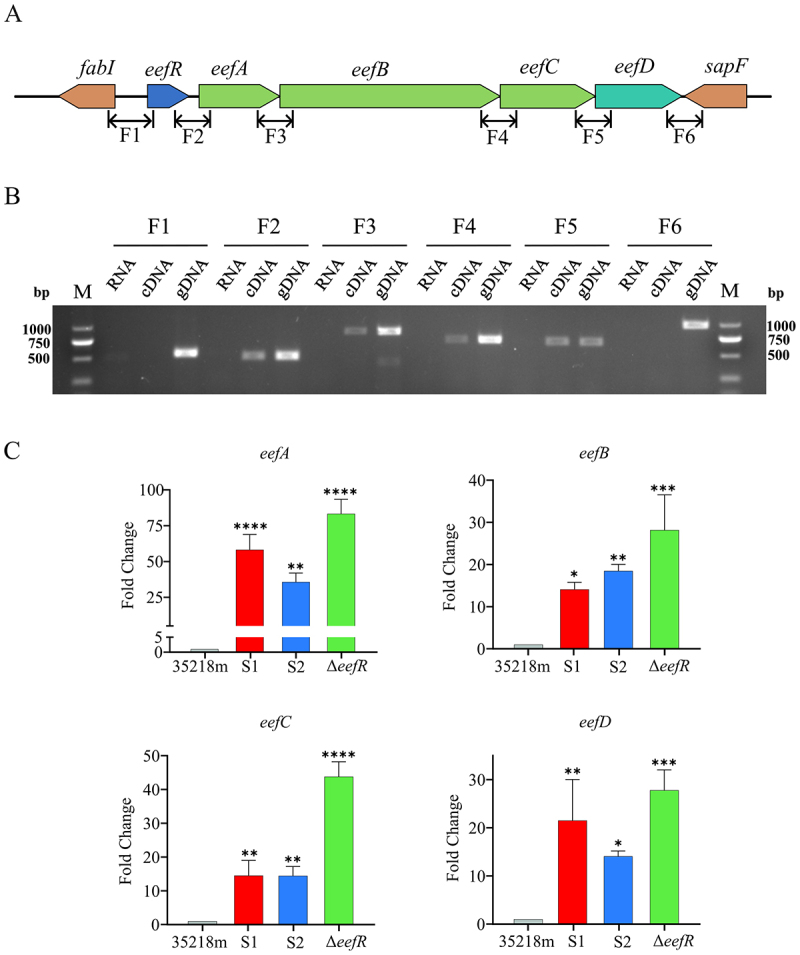
Expression analysis of the *eefR-eefABC-eefD* gene cluster. A, schematic diagram of the *eefR-eefABC-eefD* cluster. The six amplification fragments (F1 to F6) employed for the evaluation of the transcriptional unit are displayed beneath the *eef* cluster as lines. B, transcription unit determination of the *eefR* cluster. Six fragments (F1 to F6) were PCR amplified and resolved by electrophoresis. Samples using total RNA, cDNA, and genomic DNA (gDNA) as the templates are indicated. Total RNA was extracted from strain S1 cells, and cDNA was synthesized using random primers from the extracted total RNA. C, expression levels of *eefR*, *eefA*, *eefB*, *eefC* and *eefD* in 35218m, S1, S2 and Δ*eefR*.

Since the majority of TetR members are known to be regulators of their neighboring genes [25], the surrounding sequences of *eefR* were then analyzed. As illustrated in Figure 5(A) and Table S5, the *eefR* gene was oriented in the same transcriptional direction as four clustered neighboring genes *eefABCD*. Based on the sequence similarities, *eefA*, *eefB*, and *eefC* were respectively proposed to encode a periplasmic adaptor protein, an inner membrane RND-transporter, and an outer membrane channel protein, which together constitute a three-component RND-efflux pump (EefABC). In addition, *eefD* was postulated to be a putative MFS-type drug exporter gene. Gene organization and functional connectivity imply that these five genes constituted a co-transcriptional unit. As anticipated, the five intergenic regions were all successfully amplified when cDNA was employed as the template, which was reverse transcribed from total RNA in cultured cells of S1 (Figure 5(B)), indicated that the *eefR*, *eefA*, *eefB*, *eefC*, and *eefD* genes comprised an operon and were cotranscribed as a single unit.

To ascertain the impact of EefR on the expression of neighboring genes, qRT-PCR was conducted to assess the expression levels of these efflux pump genes in *eefR*-deficient mutants and their parental strains with a WT *eefR* gene. As anticipated, the destruction of *eefR* markedly enhanced the expression of the four efflux pump genes (Figure 5(C)). For example, the quantification of *eefA*, *eefB*, *eefC*, and *eefD* mRNA levels by qRT-PCR revealed respective increases of 83-fold, 28-fold, 43-fold, and 27-fold in the Δ*eefR* strain, compared to its parental counterpart 35218m. Similar upregulation patterns for these genes were observed in the S1 and S2 mutant strains relative to the parent strain. These results were consistent with the co-transcription of the *eefRABCD* operon as a single unit, as described above, and indicated that EefR functions as a negative regulator, inhibited not only its own transcription but also the expression of the four neighboring genes.

### The overproduction of EefABC and/or EefD results in multidrug resistance in *E. coli*

Alignment of the four efflux proteins with transporters from the TCDB [26] revealed that EefA, EefB, and EefC showed the highest similarity to the components of the RND efflux system EefA, EefB, and EefC in *Enterobacter aerogenes*, with sequence identities of 78.1%, 87.3% and 74.9%, respectively; While EefD exhibited the highest similarity to the MFS efflux pumps MSMEG_2991 from *Mycobacterium smegmatis* and Bcr from *E. coli*, with sequence identities of 36.7% and 30.1%, respectively. It had been demonstrated that *E. aerogenes* Δ*acrA*, expressed the EefABC efflux pump from multicopy plasmids, conferred MDR to a broad spectrum of antibiotics, including chloramphenicol, norfloxacin, ciprofloxacin, erythromycin, tetracycline, and doxycycline [27]. Similarly, the overexpression of MSMEG_2991 in *E. coli* resulted in elevated resistance toward structurally unrelated groups of antibiotics [28]. In light of these findings, it could be proposed that the overexpression of the tripartite efflux pumps EefABC and/or the MFS pump EefD might result in MDR.

To determine whether *eefABCD* could confer drug resistance, four plasmids pBR*eefABCD*, pBR*eefABC*, pBR*eefAB*, and pBR*eefD* were constructed and transformed into *E. coli* DH5α Δ*acrB*. pBR*eefD* conferred resistance to SAN at a level four times higher than the empty plasmid, and provided 4-fold resistance to chloramphenicol, tetracycline, erythromycin, and clindamycin, as well as 2-fold resistance to fusidic acid (Table 2). pBR*eefABC* conferred higher resistance than pBR*eefD*, with resistance levels of 8-fold to SAN, 32-fold to chloramphenicol, erythromycin and 16-fold to tetracycline, clindamycin, and fusidic acid. These findings indicated that the *eefABCD* operon had the potential to confer significant MDR properties to bacterial hosts. It was worth mentioning that pBR*eefABCD* conferred the same resistance pattern as pBR*eefABC*, which suggested little or no synergistic effect with the combination of *eefABC* and *eefD*. However, due to the limited number of compounds analyzed, we could not rule out the possibility that EefD in combination with EefABC might have synergistic effects or additional physiological functions. Intriguingly, pBR*eefAB* conferred a comparable or slightly diminished resistance profile relative to pBR*eefABC*, except for chloramphenicol. The MIC of chloramphenicol was 4-fold higher in *E. coli* DH5α Δ*acrB* transformed with pBR*eefAB* compared to when it carried pBR*eefABC*. The data suggested that, in addition to EefC, EefAB could form a three-component efflux pump with other OM proteins, such as TolC.

**Table 2.** Mics of sanguinarine and other antibiotics against *Escherichia coli* DH5α Δ*acrB* with different plasmids.

Compound	pBR322	pBR*eefAB*	pBR*eefABC*	pBR*eefABCD*	pBR*eefD*
Sanguinarine	1	4	8	8	4
Tetracycline	0.125	1	2	2	0.5
Erythromycin	8	128	256	256	32
Chloramphenicol	1	128	32	32	4
Clindamycin	16	256	256	256	64
Fusidic acid	16	128	256	256	32
Polymyxin B	1	1	1	1	1

**Figure 6.**
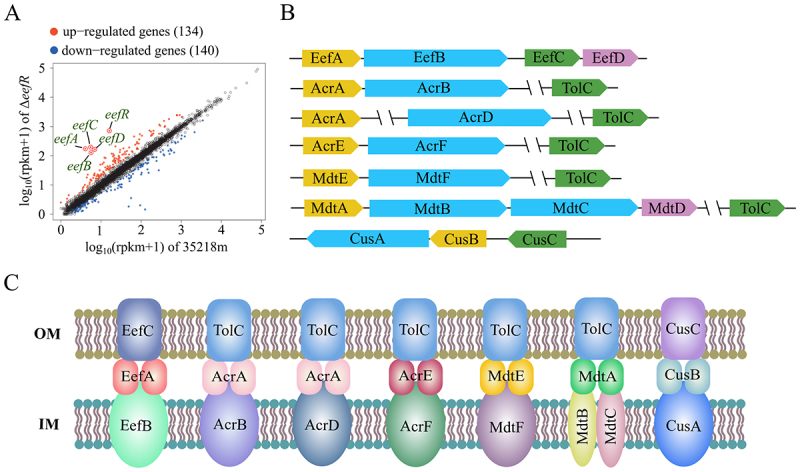
The RND efflux system in *E. coli* 35218m. A, the expression differences of genes between Δ*eefR* and 35218m. The five most significantly up-regulated genes are highlighted with red circles and labeled in green text. B, schematic diagram of seven RND efflux system in the genome of *E. coli* 35218m. “\\” indicates that the two genes are not linked in the genome. C, schematic diagram of the three-component structure of the seven RND efflux pumps located on the membrane.

To gain more information on the resistance mediated by the EefR, the transcriptomes of 35218m and Δ*eefR* were determined by RNA sequencing. With a significance threshold of log_2_ (FC) > 1.0 and a *p* value < 0.05, 274 genes were found to have altered expression levels in Δ*eefR* compared to its parental strain 35218m. As expected, the transcript levels of genes encoding the RND tripartite efflux system EefABC and the MFS pump EefD were most significantly upregulated in the Δ*eefR* strain compared to its parental strain (Figure 6(A)). Although the genome of 35218m contained seven operons for RND efflux pumps (Figure 6(B,C)), the genes encoding the other six RND efflux systems were not upregulated at all in Δ*eefR* strain. Moreover, no notable alterations were discerned in the expression levels of efflux pumps belonging to other superfamilies that contribute to MDR, with the exception of the pronounced downregulation observed in the multidrug transporters *mdtJI* and *mdtN* in the Δ*eefR* strain. These results suggested that increased expression of the *eefABCD* operon might be the primary factor contributing to the enhanced MDR observed in the *eefR*-deficient *E. coli* strain.

### The *eefRABCD* operon is widely distributed among Enterobacteriaceae bacteria

**Figure 7.**
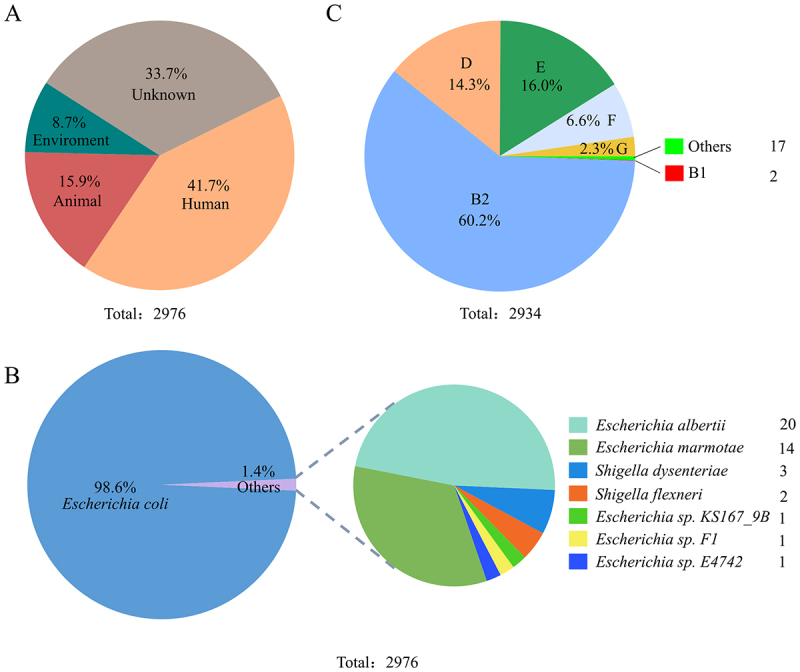
The distribution of the *eefR-eefABC-eefD* gene cluster in bacteria. A, sources of the bacteria containing the gene cluster. B, the taxonomic distribution of the bacteria containing the gene cluster. Bacterial strains other than *E. coli* were displayed in a separate pie chart with labels, where the numbers indicate the corresponding strain counts. C, phylogenetic typing of *E. coli* strains by ClermonTyping tool.

In order to analyze the distribution of the *eefRABCD* operon, BLAST was used to assemble a comprehensive data set comprising all independent isolates carrying the gene cluster that have been deposited in the GenBank database. As illustrated in Figure 7(A), a total of 2,976 isolates were found to carry the *eefR-eefABC-eefD* gene cluster, of which 41.7% were isolated from human clinical samples. From a taxonomic perspective, the isolates predominantly comprised of *E. coli* (Figure 7(B)), with 60.2% of them belonging to phylogroup B2 (Figure 7(C)). Notably, while the operon was resided on chromosome in most isolates, five *E. coli* strains harbored it on plasmids (Table S6), suggesting a plasmid-mediated transmission route.

## Discussion

**Figure 8.**
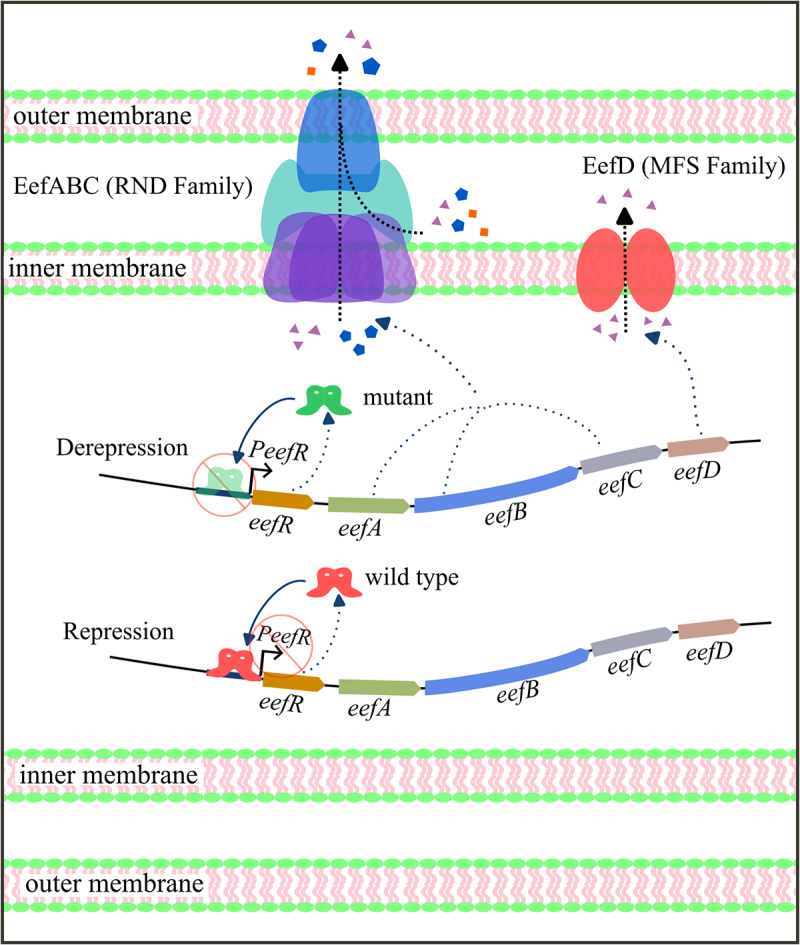
Mechanism of drug resistance mediated by TetR regulator EefR. The binding of wild type EefR to the promoter represses the expression of its own gene as well as four efflux pump genes. Lack of binding of the regulator to the intergenic regions, the gene cluster expresses the RND family efflux pump system EefABC and the MFS family efflux pump EefD, resulting in the efflux of associated substrates and conferring novel resistance capabilities to the host.

The RND pumps have been reported to exert significant influence on antibiotic susceptibility in Gram-negative bacteria. The most important and best understood efflux pump in *E. coli* is AcrAB-TolC. Overproduction of AcrAB in *E. coli* and other Enterobacteriaceae, including *Salmonella* species, has been identified as a significant mechanism of multidrug resistance in clinical isolates [29–32]. Pharmacological inhibition of the AcrAB-TolC efflux system and its homologs represents a promising strategy to counteract antibiotic resistance by restoring drug accumulation in Gram-negative pathogens [33]. While extensive screening has identified numerous efflux pump inhibitors with *in vitro* efficacy against major RND pumps, clinical translation remains unachieved. One bottleneck arises from compensatory resistance mediated by functionally redundant efflux systems. Thus, a comprehensive understanding of the adaptive evolution of *E. coli* under conditions where major RND efflux pumps are functionally defective due to spontaneous mutations or inhibited by efflux pump inhibitors is critical for combating bacterial resistance. *E. coli* strain 35218m is a spontaneous mutant with a deletion mutation in *acrB*, which arose in cultures of the ATCC 35218 strain during serial passage [9]. Compared to the wild-type *E. coli* strain 35218m exhibits significantly increased sensitivity to SAN but is prone to developing resistant revertant mutations. The present study revealed that a single exposure to SAN could induce multiple antibiotic resistance in an *acrB*-deficient strain of *E. coli* ATCC 35218. Detailed research had revealed that the primary cause of this resistance might be the overexpression of the cryptic multidrug efflux pumps EefABC and EefD, resulting from mutations in EefR, a novel negative TetR regulator (Figure 8).

The *eefABC* genes were initially identified in *E. aerogenes* [27], although this cluster lacked *eefR* and *eefD*. While *eefR* was first reported in 2008 to be clustered with efflux system *eefABCD* in the genome of *E. coli* SMS-3–5 [34], EefR’s biological function has remained experimentally uncharacterized since then. Our study now provides the first mechanistic evidence that EefR functions as a repressor of its own and *eefABCD* expression. EefR represents a paradigm-shifting addition to the TetR family, distinguished by its divergent regulatory logic and evolutionary path. Despite it shares 21% sequence identity with UidR, a β-glucuronidase repressor [18,35], EefR has functionally diverged to control a cryptic RND/MFS efflux operon (*eefABCD*) that is absent from canonical *E. coli* resistance networks. RNA-seq data confirmed that EefR exclusively activates *eefABCD* without upregulating other efflux systems. This functional repurposing is underscored by its unique TGAGAAN5TTCTCA binding motif, which resembles no other characterized TetR recognition sequences [36–47]. Crucially, while the homologous EefABC pump in *E. aerogenes* is silenced by H-NS [27], EefR autonomously regulates *eefABCD* in *E. coli*, reflecting a lineage-specific adaptation. Taken together, EefR’s unique operon specificity and DNA binding code solidify its status as a novel TetR variant, expanding the functional scope of the family in bacterial stress adaptation.

Prior to this study, six systems belonging to the RND family had been functionally described in *E. coli*: AcrAB-TolC, AcrAD-TolC, AcrEF-TolC, MdtABC-TolC, MdtEF-TolC, and CusABC, all of which were found in the genome of strain 35218m (Figure 6(B,C)). The results of this study have led to the characterization of EefABC from strain 35218m, which represents the seventh RND-type efflux pump identified in *E. coli*, adding to the known MDR pumps in this bacterium. In addition, *eefABC* is found to be prevalent in group B2 *E. coli* strains but absent from the genome of the commonly used laboratory strain K-12 (group A). Notably, while this manuscript was in preparation, Pugh and colleagues had already reported in a bioRxiv preprint that EefABC, as the seventh RND efflux system, is widely distributed in pathogenic *E. coli* genomes and characterized the functions of EefABC and EefD [48]. However, their study, based on gene knockout and overexpression experiments, concluded that neither deletion nor overexpression of EefABCD alters antimicrobial susceptibility, which is inconsistent with our findings.

The observed discrepancies between our experimental results and Pugh et al.’s preprint findings regarding EefABC/EefD-mediated antimicrobial susceptibility changes in *E. coli* Δ*acrB* mutants may stem from multiple technical and biological factors: (1) genetic divergence in pump sequences between clinical isolates, (2) vector systems, (3) host backgrounds, and (4) cis-regulatory requirements. Crucially, our data demonstrate that *eefA* upstream sequences are essential for conferring drug susceptibility phenotypes when expressing multi-gene operons (*eefAB/ABC/ABCD*), whereas individual *eefB* or *eefD* structural genes alone suffice to alter susceptibility, suggesting operon architecture and putative native promoter elements within *eefA* 5’ regions may orchestrate proper pump assembly/function or interact with unidentified transcriptional regulators absent in minimal expression systems. These findings underscore the context-dependence of RND pump characterization and highlight that standardized genetic contexts (including native regulatory regions) are critical for reconciling functional studies of multidrug efflux systems.

In conclusion, this study demonstrates that mutations in the previously uncharacterized TetR family repressor EefR, which acts via its unique binding motif, trigger overexpression of the cryptic efflux pump operon *eefABCD* and thereby confer multidrug resistance in AcrB-deficient *E. coli*, particularly in response to SAN. These findings illuminate key adaptive strategies that microbes use to survive antimicrobial pressure. They also underscore potential environmental risks linked to SAN-containing products, whose widespread use may facilitate the spread of such resistance genes, though further research is needed to clarify their long-term ecological impacts.

## Supplementary Material

supplement 1.docx

## Data Availability

The data that support the findings of this study are openly available in figshare at https://doi.org/10.6084/m9.figshare.28682663.v8, reference number [49].
