# Supplementary material for: EefR mutations drive sanguinarine resistance by activating cryptic multidrug efflux pumps in AcrB-Null Escherichia coli
Source: Virulence. 2025 Sep 29;16(1):2566244. doi: 10.1080/21505594.2025.2566244 (PMC12482432; doi:10.1080/21505594.2025.2566244)
Supplement: supplement 1.docx [file KVIR_A_2566244_SM3550.docx]

**Table S1** Strains and plasmids used in this study.

| **Strains or plasmids** | **Genotype or description** | **Sources** |
| --- | --- | --- |
| **strain** |  |  |
| *E. coli* ATCC 35218 | Wild type strain | ATCC* |
| 35218m | *E. coli* ATCC 35218 Δ*acrB* (spontaneous mutant), parental strain of this work. | [1] |
| *E. coli* DH5α | F– φ80lacZΔ M15 Δ (*lacZYA-argF*) *U169 recA1 endA1 hsdR17* (rK^–^, mK^+^) *phoA supE44* λ- *thi–1 gyrA96 relA1* | Stratagene** |
| *E. coli* DH5α Δ*acrB* | *E. coli* DH5α *acrB*::*kan*; Km^R^ | [1] |
| *E. coli* BL21 (DE3) | F– *omp*T *hsdS_B_* (rB^–^, mB^–^) *gal dcm* (DE3) | Sangon Biotech† |
| Δ*eefR* | 35218m Δ*eefR* | Knockout via CRISPR-Cas system, see Methods 2.4 |
| CΔ*eefR* | Δ*eefR* containing plasmid pBR-*eefR*; Tc^R^ | *eefR* complemented strain, see Methods 2.4 |
| S1 | Sanguinarine-induced spontaneous resistant mutant derived from 35218m | Spontaneous resistant mutant, see Methods 2.3 |
| S2 | Sanguinarine-induced spontaneous resistant mutant derived from 35218m | Spontaneous resistant mutant, see Methods 2.3 |
| S1-C*eefR* | S1 containing plasmid pBR-*eefR*; Tc^R^ | *eefR* complemented strain, see Methods 2.4 |
| S2-C*eefR* | S2 containing plasmid pBR-*eefR*; Tc^R^ | *eefR* complemented strain, see Methods 2.4 |
| **Plasmids** |  |  |
| pET28a (+) | T7 expression vector; Km^R^ | Miaoling Bio †† |
| pET28a-*eefR* | pET28a (+)::*eefR*; Km^R^ | Constructed using Gibson Assembly, see Methods 2.6 |
| pCas | *repA101* (Ts) Km^R^ *Pcas*-*cas9 P_araB_*-*Red lacI^q^ P_trc_*-*sgRNA*-*pMB1* | [2] |
| pTargetF | pMB1-sgRNA; Spc^R^ | [2] |
| pTargetF (*tetR*) | pTargetF Δ*smR*::*tetR*, Tc^R^ | Constructed using Gibson Assembly, see Methods 2.4 |
| pTargetF-*eefR* | pTargetF (*tetR*) derivative carring sgRNA-*eefR* | Constructed using Gibson Assembly, see Methods 2.4 |
| pBR322 | pMB1; Ap^R^, Tc^R^ | Takara‡ |
| pBR*eefR* | pBR322 Δ*ampR*::*eefR*; Tc^R^ | Constructed using Gibson Assembly, see Methods 2.4 |
| pBR*eefAB* | pBR322 Δ*tetR*::*eefAB*; Ap^R^ | Constructed using Gibson Assembly, see Methods 2.9 |
| pBR*eefABC* | pBR322 Δ*tetR*::*eefABC*; Ap^R^ | Constructed using Gibson Assembly, see Methods 2.9 |
| pBR*eefABCD* | pBR322 Δ*tetR*::*eefABCD*; Ap^R^ | Constructed using Gibson Assembly, see Methods 2.9 |
| pBR*eefD* | pBR322 Δ*tetR*::*eefD*; Ap^R^ | Constructed using Gibson Assembly, see Methods 2.9 |
| pBR-*P_0_*-GFP | pBR322 ::*gfp*; Ap^R^, Tc^R^ | Constructed using Gibson Assembly, see Methods 2.8 |
| pBR-*P_eefR_* -GFP | pBR322 ::*P_eefR_*::*gfp*; Ap^R^, Tc^R^ | Constructed using Gibson Assembly, see Methods 2.8 |
| pBR-*P_eefR_* -GFP-EefR | pBR-*P_eefR_* -GFP Δ*tetR*::*eefR*; Ap^R^ | Constructed using Gibson Assembly, see Methods 2.8 |
| pBR-*P_eefR_* -GFP-EefR ^S1^ | pBR-*P_eefR_* -GFP Δ*tetR*::*eefR* (S1); Ap^R^ | Constructed using Gibson Assembly, see Methods 2.8 |
| pBR-*P_eefR_* -GFP-EefR ^S2^ | pBR-*P_eefR_* -GFP Δ*tetR*::*eefR* (S1); Ap^R^ | Constructed using Gibson Assembly, see Methods 2.8 |

Note: Km^R^, kanamycin resistant; Tc^R^, tetracycline resistant; Spc^R^, spectinomycin resistant; Ap^R^, ampicillin resistant.

*****, ATCC, American Type Culture Collection, Manassas, USA.

**, Stratagene, Agilent Technologies, Santa Clara, USA.

†, Sangon Biotech, Shanghai, China.

††, Miaoling Bio, Wuhan Miaoling Biotechnology Co., Ltd., Wuhan, China.

‡, Takara, Takara Bio Inc., Kusatsu, Japan.

**Table S2** Primers used in this study.

|  | **Relative description** | **Usages** |
| --- | --- | --- |
| **Primers** | **Sequence (5′→ 3′)** |  |
| ssDNA | GCTAAGCTGAGCTTCGCTGGCAATTTGCGACATACTTTATT***ATTAAT***TGGCATGAAATCCACAGAGACGAAAACACCGACGGGCGGCGTT | Homologous fragment for knockout |
| pTargetF- F | GCTGGCAATTTGCGACATACGTTTTAGAGCTAGAAATAGCAAG | Forward primer to construct pTargetF-*eefR* |
| pTargetF- R | GTATGTCGCAAATTGCCAGCACTAGTATTATACCTAGGACTGAG | Reverse primer to construct pTargetF-*eefR* |
| pBR-*eefR*-F | GAGTAAACTTGGTCTGACAGCCGCTATTGGTTAATTCTGT | Forward primer to amplify *eefR* gene carrying pBR322 homologous fragment |
| pBR-*eefR*-R | AATATTGAAAAAGGAAGAGTATGACCAGCAAGCTGGAG | Reverse primer to amplify *eefR* gene carrying pBR322 homologous fragment |
| pBR-f | ACTCTTCCTTTTTCAATATTATTG | Forward primer to amplify pBR322 plasmid vector |
| pBR-r | CTGTCAGACCAAGTTTACTC | Reverse primer to amplify pBR322 plasmid vector |
| pET-*eefR*-F | CTTTAAGAAGGAGATATACCATGACCAGCAAGCTGGAG | Forward primer to amplify *eefR* gene carrying pET28a (+) homologous fragment |
| pET-*eefR*-R | CTCAGTGGTGGTGGTGGTGGTGTTTATTTATCAACATACTAACAATATCCTG | Reverse primer to amplify *eefR* gene carrying pET28a (+) homologous fragment |
| pET-F | CACCACCACCACCACCACTGAG | Forward primer to amplify pET28a (+) plasmid vector |
| pET-R | GGTATATCTCCTTCTTAAAGTT | Reverse primer to amplify pET28a (+) plasmid vector |
| *eefR*-gel-F | CACAATCTCTTCATTATCAG | Forward primer to synthesize *eefR* promoter region probes |
| *eefR*-gel-R | TTCTGTCTCCTGGCCTATC | Reverse primer to synthesize *eefR* promoter region probes |
| *eefR*-gel-Flu | CACAATCTCTTCATTATCAG | Forward primer to synthesize *eefR* promoter region probes with biotin |
| *eefA*-gel-F | ACAGAATTAACCAATAGCGG | Forward primer to synthesize nonspecific probes |
| *eefA*-gel-R | TATTTTCTCCAGGGTGAGAAA | Reverse primer to synthesize nonspecific probes |
| *eefR* -del-R | TTTTTGCTAATTATACGTTTCTCC | Reverse primer to synthesize probes with deletions |
| MT-1-R | TTCTGTCTCCTGGCCTATCACTTGGTGAGGATGATCGTTCTCATTTTTGC | Reverse primer to synthesize *eefR* promoter region probes with a base mutation |
| MT-2-R | TTCTGTCTCCTGGCCTATCACTTGGTGAAGATGATCGTTCTCATTTTTGC | Reverse primer to synthesize *eefR* promoter region probes with two base mutations |
| MT-4-R | TTCTGTCTCCTGGCCTATCACTTGGTGGAGGTGATCGTTCTCATTTTTGC | Reverse primer to synthesize *eefR* promoter region probes with four base mutations |
| MT-6-R | TTCTGTCTCCTGGCCTATCACTTGGCAGAGGTGATCGTTCTCATTTTTGC | Reverse primer to synthesize *eefR* promoter region probes with six base mutations |
| pBR-F1 | AAGATGGGGATGCATTCTGCCGAGGATGACGATGAG | Forward primer to amplify pBR322 plasmid vector carrying *eefA* homologous fragment |
| pBR-R1 | TGTTGCGTCGTTCTCTTCCTATACCTTGTCTGCCTC | Reverse primer to amplify pBR322 plasmid vector carrying *eefA* homologous fragment |
| *eefA*-F | AGAATGCATCCCCATCTTAG | Forward primer to amplify *eefAB* gene |
| *eefB*-R | AAGAGAACGACGCAACAT | Reverse primer to amplify *eefAB* gene |
| pBR-R2 | GGCATGTCATCACTCAAGTAACCTATACCTTGTCTGCCTC | Reverse primer to amplify pBR322 plasmid vector carrying *eefB* homologous fragment |
| *eefC*-R | TTACTTGAGTGATGACATGCC | Reverse primer to amplify *eefABC* gene |
| pBR-R3 | CTATACCTTGTCTGCCTC | Reverse primer to amplify pBR322 plasmid vector carrying *eefC* homologous fragment |
| *eefD*-R1 | GGGAGGCAGACAAGGTATAGTTACTTTTGATTGTCGATTCTGG | Reverse primer to amplify *eefABC* gene carrying *eefABCD* homologous fragment |
| pBR-F2 | AAGAGAGACTCTAGCCATGCCGAGGATGACGATGAG | Forward primer to amplify pBR322 plasmid vector carrying *eefD* homologous fragment |
| pBR-R4 | ACAATCAAAAGTAAGGAACCTATACCTTGTCTGCCT | Reverse primer to amplify pBR322 plasmid vector carrying *eefD* homologous fragment |
| *eefD*-F | ATGGCTAGAGTCTCTCTTTC | Forward primer to amplify *eefD* gene |
| *eefD*-R | TTCCTTACTTTTGATTGTCGAT | Reverse primer to amplify *eefD* gene |
| *gfp*-F | CTTGCGGAGAACTGTGAATGTTATTTGTAGAGCTCATCCATGC | Forward primer to amplify *gfp* gene carrying pBR322 homologous fragment |
| *gfp*-R | ATGAGTAAAGGAGAAGAACTT | Reverse primer to amplify *gfp* gene |
| pBR-GFP-F | CATTCACAGTTCTCCGC | Forward primer to amplify pBR322 plasmid vector |
| pBR-GFP-R | CGCAAACCAACCCTTG | Reverse primer to amplify pBR322 plasmid vector |
| *P_eefR_*-F | AGTTCTTCTCCTTTACTCATTTCTGTCTCCTGGCCTATC | Forward primer to amplify *PeefR* gene carrying *gfp* homologous fragment |
| *P_eefR_*-R | CTGCCAAGGGTTGGTTTGCGTTGCGTTATCTACTCCTGACG | Reverse primer to amplify *PeefR* gene carrying pBR322 homologous fragment |
| *eefR*-F2 | ATGACCAGCAAGCTGG | Forward primer to amplify *eefR* gene |
| *eefR*-R2 | TTATTTATTTATCAACATACTAACAATATCC | Reverse primer to amplify *eefR* gene |
| pBR-GFP-f | ATCTCCAGCTTGCTGGTCATACACGGTGCCTGACT | Forward primer to amplify pBR-P*eefR*-GFP plasmid vector carrying *eefR* homologous fragment |
| pBR-GFP-r | GTATGTTGATAAATAAATAAATGGAAGCCGGCG | Reverse primer to amplify pBR-P*eefR*-GFP plasmid vector carrying *eefR* homologous fragment |
| S1-F | TTAGCGAGGGTCAGATATATCGTTACTTCGCC | Forward primer to construct pBR-*PeefR*-GFP-EefR^S1^ |
| S1-R | ATATATCTGACCCTCGCTAAGCTGAGCTTCGCT | Reverse primer to construct pBR-*PeefR*-GFP-EefR^S1^ |
| S2-F | GGCAATGCTTGAGAAGCTGACGCCAGAATG | Forward primer to construct pBR-*PeefR*-GFP-EefR^S2^ |
| S2-R | TCAGCTTCTCAAGCATTGCCATAACCCG | Reverse primer to construct pBR-*PeefR*-GFP-EefR^S2^ |

Note: Homologous segments are underlined. The *AseI* restriction site in ssDNA is indicated in bold italics.

**Table S3** qRT-PCR Primers used in this study.

|  | **Relative description** | **Usages** |
| --- | --- | --- |
| **qRT-PCR Primers** | **Sequence (5′→ 3′)** |  |
| 16s rRNA-f | GCTAATACCGCATAACGTCG | Forward primer to amplify 16s gene |
| 16s rRNA-r | TCATCCTCTCAGACCAGCTA | Reverse primer to amplify 16s gene |
| *eefR*-f | TTGAAGAGATGGTTCGCCG | Forward primer to amplify *eefR* gene |
| *eefR* -r | TGTGCTCGTCGCTAAGAT | Reverse primer to amplify *eefR* gene |
| *eefA*-f | CAATGCTGCTCTTATCGGG | Forward primer to amplify *eefA* gene |
| *eefA*-r | CATCAATCTGGTAGAGAGGC | Reverse primer to amplify *eefA* gene |
| *eefB*-f | TTATACTGGTGCTTCTGCCG | Forward primer to amplify *eefB* gene |
| *eefB*-r | TTCTGTACCTGAACCTGTGC | Reverse primer to amplify *eefB* gene |
| *eefC*-f | AATGCGGCCTTAAGTTCGAC | Forward primer to amplify *eefC* gene |
| *eefC*-r | AGCAATCAGCGTTAAACGAGT | Reverse primer to amplify *eefC* gene |
| *eefD*-f | GCAATTAACCCTGACCGCATC | Forward primer to amplify *eefD* gene |
| *eefD*-r | TCCCTTGAATAAAACGCCAGACC | Reverse primer to amplify *eefD* gene |

**Table S4** Gene *eefR* in mutant stains of five additional SAN-resistant isolates compared to the parent strain.

| **Strains** | **Genome position** | **Gene** | **Mutation** | **Change** | **Description** |
| --- | --- | --- | --- | --- | --- |
| S3 | 2770431 | BGPOLHDB_02578 | C-G | Missense variant (Val45Glu) | HTH-type transcriptional regulator |
| S4 | 2770396 | BGPOLHDB_02578 | AAGCTG-A | Frameshift variant | HTH-type transcriptional regulator |
| S5 | 2770109 | BGPOLHDB_02578 | TG-T | Frameshift variant | HTH-type transcriptional regulator |
| S6 | 2770034 | BGPOLHDB_02578 | A-C | Stop gained  (Leu 164 Stop) | HTH-type transcriptional regulator |
| S7 | 2770426 | BGPOLHDB_02578 | A- CGACATA | Frameshift variant | HTH-type transcriptional regulator |

**Table S5** The*eefRABCD*gene cluster and its upstream 316 bp sequence.

| **Sequence (5′→ 3′)** |
| --- |
| TTGCGTTATCTACTCCTGACGCCTGCAAGGGGGAGGTTGGGGAAATATCTTATAGAATCTGACTTTGAAACTATTCGTATTAGCTCCGATAGGTTTGATGATGAAAACAGAACTGGCGACAACAAGTCGCTGTCTTTCTGATGAATTGACCCCAACTTAATATTGTCAACTATCTTCACAGAATCTCCACAATCTCTTCATTATCAGAATATGCATCATGAATAAGATAAAAATACTTTCCATTACTTTGGAGAAACGTATAATTAGCAAAAATGAGAACGATCATTCTCACCAAGTGATAGGCCAGGAGACAGAAATGACCAGCAAGCTGGAGATACGCCACAAACAGCGTCAGGATGAAATCATTAACGCCGCCCGTCGGTGTTTTCGTCTCTGTGGATTTCATGCCGCCAGTATGTCGCAAATTGCCAGCGAAGCTCAGCTTAGCGTGGGTCAGATATATCGTTACTTCGCCAACAAAGACGCCATTATTGAAGAGATGGTTCGCCGTATCATCGATTTTCGTATTGCCCAAATGGACATTGACGCGCGTACCGATCACTTCCCGGAAGTCCTTGCCTTGCGTAAATCCTTAAATGAGGACGACGACGCGCTGATGCTGGAAGTGGCTGCAGAAGCCACACGCAACCCGCGGGTTATGGCAATGCTTGAAGAAGCTGACGCCAGAATGTTCGCTAACGGTTGTGCACATATGAAAAGAATGCATCCCCATCTTAGCGACGAGCACATTCGTTGCTGCGTTGAAGTGTTTGCTACCATGATGGAAGGTACGGTTTATCGTCGTTTAACACCGCAAAAAAGCGATCCACAACATCTGCAGGAAATATATCAGGATATTGTTAGTATGTTGATAAATAAATAAGAACAGAATTAACCAATAGCGGGGGATATTAATCCCCCTTATTTCATTGCGAAATTCATTTCGCGATGCGGGTTCGTTATAACTGAATATCCCTGACATATCCTCATTAATTTCAATGAGGAGGGTATTTCTCACCCTGGAGAAAATAATGAAGTATATAGCAACATCTGTAGTGGCAATGCTGCTCTTATCGGGTTGTGATAATACGCAAAGTAACAATTCATCCCCGTCAGAAACAGAAGTCGGCGTTGTTACGCTCAAATCTCAACCGGTTTCGGTAGTCAGTGAATTAACCGGACGCACCAGTGCTGCGCTCAGTGCTGAAGTACGTCCGCAGGTTGGGGGAATTATCCAGAAACGCTTATTTAAGGAAGGTGATCTGGTCAAGGCTGGACAGCCTCTCTACCAGATTGATGCGGCCAGTTATCAGGCTGCATGGAATGAAGCCCGGGCAGCATTACAACAAGCACAGGCACTGGTAAAAGCCGATTGCCAGAAAGCGCAGCGTTATGCCCGACTGGTGAAAGAGAACGGTGTTTCACAACAGGATGCTGATGATGCTCAGTCTACCTGTGCACAAGATAAAGCCAGTGTAGAGGCGAAAAAAGCCGCACTGGAAACTGCGCGCATTAATCTTGACTGGACCACGGTAACCGCACCGATTTCGGGGCGTATTGGCATTTCGTCGGTAACCCCTGGCGCACTGGTGACCGCGTCGCAGGATACAGCGTTAACGACTATTCGTGGTCTGGATACAATGTATGTCGACCTCACTCGCTCCAGTGTCGATTTATTACGTCTGCGTAAACAGTCACTGGCGACCAACAGTGACACTATGAGCGTCTCACTTATTCTGGAAGATGGCACAACCTACAGCGAAAAAGGGCGTCTGGAACTCACCGAAGTCGCGGTGGATGAGTCTACCGGTTCGGTGACATTACGGGCAATTTTCCCCAATCCACAACAGCAGTTATTACCGGGAATGTTTGTTCGCGCTCGTGTCGATGAAGGCGTGATGGAAAACGCTATTCTCGCGCCTCAACAGGGCGTTACGCGCGATGCTAAAGGCAATGCAACTGCGCTGGTGGTGAATAAAGACAATAAAGTAGAGCAGCGAACGCTCGAAACGGGAGAAACGTATGGTGATAAATGGCTGGTGCTGAACGGCCTGCACAACGGCGACCGACTGATTGTTGAAGGTTCTGCCAAAGTCACTTCAGGGCAGACCGTCAAGGCTGTTGAAGTTCAGGCTAATGGAGGCAACGCCTGATGTTTTCGCGCTTTTTTGTTCGCCGCCCGGTCTTTGCCTGGGTTATCGCCATTTTGATTATGTTGGCGGGGATTCTGGCTATTCGCACATTGCCCGTCGCGCAATATCCTGACGTTGCACCGCCGACCATTAAAATTTCAGCCACTTATACTGGTGCTTCTGCCGAAACGCTGGAGAACAGCGTGACTCAGGTTATCGAGCAACAACTCACTGGGCTTGATAATTTACTCTATTTCAGCTCAACCAGTAGCTCTGATGGTTCGGTCAGTATTAATGTGACCTTTGAACAAGGTACCGATCCAGATACTGCACAGGTTCAGGTACAGAATAAAATTCAGCAGGCGGAGTCGCGCCTACCCAGCGAAGTGCAGCAAACGGGTGTTACGGTGGAGAAATCACAAAGCAACTTTTTGCTGATTGCTGCCGTGTATGACACCACTGACAAAGCCTCCAGTTCGGATATCGCCGACTGGCTGGTCAGTAACGTTCAGGACCCGCTGGCGCGTGTTGAAGGTGTGGGGAGTCTGCAAGTCTTTGGCGCGGAATACGCTATGCGCATCTGGCTTGACCCGGCCAAACTGGCGTCTTACTCGCTGATGCCTTCAGACGTGCAAAGTGCTATTGAAGCGCAAAACGTGCAGGTTACTGCCGGGAAAATAGGGGCATTGCCTTCACCGAATACTCAGCAACTGACCGCAACGGTACGTGCGCAGTCTCGTTTGCAGACGGTGGATCAGTTCAAAAATATTATCGTGAAAAGCCAGTCAGACGGCGCAGTTGTTCGTATAAAAGATGTGGCTCGCGTTGAGATGGGCAGTGAAGATTATACCGCTATCGGCAAACTTAACGGTCACCCGTCTGCCGGGGTTGCTGTAATGCTTTCGCCCGGTGCGAATGCGCTGAATACGGCGACGCTGGTCAAGGATAAGATTGCCGAATTCCAGCGGAACATGCCGCAGGGATACGACATCGCATACCCGAAAGACAGCACTGAATTTATTAAAATCTCCGTAGAGGACGTAATTCAGACGCTGTTTGAAGCCATCGTATTGGTGGTTTGCGTGATGTATTTATTCCTGCAAAACCTGCGTGCCACACTGATTCCGGCGTTAGCTGTCCCCGTCGTTTTGCTGGGCACATTCGGCGTTCTTGCGTTGTTTGGCTATTCGATTAATACCCTGACGTTGTTTGCAATGGTGCTGGCGATCGGCTTGCTGGTGGACGATGCCATTGTGGTGGTAGAGAACGTCGAACGCATTATGCGTGACGAAGGGTTACCCGCGCGTGAAGCCACGGAAAAATCAATGGGCGAGATTTCTGGCGCACTGGTTGCCATTGCGCTGGTGTTGTCAGCCGTATTCCTGCCGATGGCCTTCTTTGGGGGATCTACGGGGGTAATTTATCGTCAGTTCTCCATCACCATCATCTCCGCAATGCTGCTTTCCGTGGTGGTGGCATTGACCTTGACTCCCGCCCTGTGCGGTTCCGTCCTCCAGCATGTTCCGCCACATAAAAAAGGCTTTTTCGGCGCATTTAACCGCTTTTACCGCCGTACTGAAGATAAATATCAGCGAGGCGTAATTTATGTCCTGCGCCGTGCAGCCCGAACGATGGGGCTTTATGTCGTGCTGGGTGGTGGAATGGCCCTGATGATGTGGAAACTGCCGGGCAGTTTCTTACCCACTGAAGACCAGGGCGAAATCATGGTGCAGTACACGCTGCCGGCCGGTGCAACCGCTGCCCGTACAGCAGAAGTGAATCGCCAGATTGTTGACTGGTTCCTGATTAACGAGAAAGCAAATACCGATGTCATCTTTACCGTTGATGGTTTCAGTTTTAGCGGCAGCGGACAGAACACCGGGATGGCGTTTGTTTCGTTGAAAAACTGGTCTCAACGTAAAGGGGCAGAAAACACCGCCCAGGCTATCGCCCTACGGGCAACCAAAGAGCTGGGCACAATTCGTGATGCCACGGTATTTGCGATGACGCCGCCAGCCGTTGATGGGCTGGGGCAAAGCAATGGTTTTACGTTTGAATTGTTAGCTAACGGTGGAACCGATCGTGAAACACTGCTGCAAATGCGTAATCAGTTGATAGAAAAAGCGAATCAAAGTCCGGAGTTACATTCTGTACGCGCCAATGATTTGCCACAAATGCCGCAATTGCAGGTAGATATTGATAGTAATAAAGCGGTGTCATTAGGGTTGAGTTTGAATGATGTCACCGACACCCTGTCCAGTGCGTGGGGCGGTACTTATGTGAATGACTTTATTGACCGTGGTCGTGTGAAAAAAGTCTACATTCAGGGCGACAGCGAATTTCGTTCCGCGCCGTCAGACTTAGGTAAATGGTTTGTGCGCGGTAGCGATAACGCCATGACACCATTCTCTGCTTTTGCGACCACCCGCTGGCTGTATGGACCGGAAAGACTGGTGCGCTACAACGGCTCGGCAGCCTATGAAATTCAGGGCGAAAACGCGACTGGCTTTAGTTCCGGCGATGCAATGACAAAAATGGAGGAACTGGCAAACAGTCTTCCTGCGGGAACAACCTGGGCCTGGAGTGGTTTGTCATTGCAGGAGAAACTGGCCAGCGGTCAGGCATTAAGTCTGTATGCAGTTTCTATTCTGGTGGTTTTCCTCTGCCTTGCAGCACTGTACGAAAGCTGGTCAGTCCCGTTCTCGGTCATTCTGGTGATCCCTCTGGGGCTTCTTGGCGCGGCGCTGGCGGCCTGGATGCGTGATTTAAACAACGACGTTTACTTCCAGGTAGCGCTATTAACTACTATCGGCTTGTCGTCGAAAAACGCCATTCTGATTGTGGAATTTGCTGAAGCGGCGGTTGCAGAGGGCTATTCCTTGAGTCGTGCGGCATTACGCGCGGCGCAGACTCGTTTACGCCCAATCATCATGACCTCGCTGGCGTTTATTGCGGGGGTAATGCCGCTGGCGATAGCAACCGGCGCAGGGGCGAACAGCCGCATCGCCATTGGTACGGGCATTATTGGCGGCACGCTGACCGCTACATTACTGGCCATTTTCTTCGTTCCTCTGTTTTTTGTACTGGTGAAACGTTTGTTTGCCGGTAAACCGCGCCGTCAGGAGTAAGTTATGTTGCGTCGTTCTCTTATTTTTCTGGTGTTGTTGAGTGCGGGATGTGTCTCGCTCGATCCTCACTACAGTACGCCTGAATCCCCAATCCCGGCGACCTTGCCGGGAGCACAGGGCCAGGGGAAGGCAATCAGCCATGACTGGCAGCAGGTGATTCACGATCCCCGATTGCAGCAGGTAGTAACCATTGCACTGAACAGTAACCGCGATGTGCAAAAAGCGATTGCTGATATTGACTCAGCACGGGCGCTCTATGGGCAAACTAATGCGTCGTTATTTCCGACGGTGAATGCGGCCTTAAGTTCGACTCGCAGTCGTTCGCTAGCGAATGGTACGGGAACAACTGCCGAGGCTGACGGCACGGTGTCCAGTTATACGTTAGATCTGTTTGGTCGTAATCAGAGTTTATCTCGGGCGGCGCGGGAAACCTGGCTTGCCAGTGAATTTACAGCCCAGAACACTCGTTTAACGCTGATTGCTGAAATCAGCACCGCCTGGCTGACACTGGCGGCCGATAACAGCAACCTGGCCCTGGCAAAAGAAACGATGGCCAGTGCAGAAAACTCATTGAAAATTATCCAGCGCCAGCAACAGGTTGGCACGGCGGCAGCGACAGATGTCAGTGAGGCGATGAGTGTTTATCAGCAAGCGCGCGCCAGCGTTGCCAGCTATCAGACACAAGTGATGCAGGATAAAAATGCGCTGAACTTACTGGCAGGTACAACGCTTGCGGAAAATCTGCTACCGGGAACACTGGAAAGCCTGCCGGAACAAATGATAAGTCTGGTTCCTGCGGGAGTATCATCTGATGTTCTGCTGCGTCGCCCGGATATCCAGGAAGCTGAACATAATTTGAAGAGCGCCAATGCAGATATTGGCGCGGCGCGGGCTAACTTTTTCCCGACGATTTCGCTGACTGCCAGTGCCGGTGTTGGCAGTGACGCATTGTCGTCGTTGTTCAGCCACGGAATGCAGATCTGGTCATTTGCGCCATCGGTCACATTACCGCTGTTCACTGGCGGAAGTAATCTGGCGCAGCTTCGTTACGCAGAAGCACAAAAGCGTGGGCTCATTGCCACCTATGAAAAAACCGTTCAGAGCGCATTTAAAGATGTCGCTAACGCACTGGCGCGGCGGACCACGCTTGAAGAGCAACTGGATGCACAGCGCCAGTATGTTAAAGCTGAGCAACAAACGGTCGATGTTGGCTTGCGTCGCTATCAGGCGGGTGTTGGTGATTACCTGACCGTGTTGACGGCGCAGCGCAGCTTGTGGAGTGCACAGCAGGAGCTGCTGGCACTGCAACTGACTGATTTTACAAACCGAATCACGCTCTGGCAGTCGCTGGGCGGCGGCATGTCATCACTCAAGTAAGGAATAATATGGCTAGAGTCTCTCTTTCATGGGCATTGATTCTTGGTCTTTTAGCCGGTATCGGCCCGATGTGTACCGATCTTTATTTGCCGGCTTTGCCGGAGATGTCTGAGCAACTGGCGGCAACCACGACCATAACGCAATTAACCCTGACCGCATCACTGATTGGTCTTGGCGTCGGACAACTGTTATTTGGCCCTCTGAGCGACAAAATAGGGCGTAAACGTCCGTTAATCTTGTCGTTGTTATTGTTTATTGTTTCTTCCATTTTGTGCGCGACAACGAACAATATTTACTGGCTGGTGGTCTGGCGTTTTATTCAAGGGATCGCGGGGGCGGGTGGTTCGGTGCTCTCTCGTTCTATTGCTCGTGACAAATATCAGGGAGTAACGTTGACCCAGTTTTTTGCGCTGTTAATGACGGTGAATGGCCTGGCACCGGTGTTGTCGCCAGTGCTGGGCGGGTACATTGTCAGCACTTTTGACTGGCGCACTTTATTCTGGGTAATGGCTGAAATTAGCACCGTACTGTTGCTGGGCTGCCTGTTATTTATTAATGAGACCTTGCCAGAAAATAAAAGGGGCTCATCATTGCTATTAACCGGACGAAGCGTGGTGCAGAACCGCCGTTTTATGCGCTTTTGCCTGATTCAAAGTTTTATGCTGGCCGGTTTGTTTGCATATATCGGCTCTTCGTCGTTCGTGTTGCAGAAGGAATTTGGCTTTAGTCCAATGCAATTTAGCCTGGTGTTTGGCCTTAACGGCATCGGACTTATCATTGCTTCATGGATCTTCTCGCGCCTGGCGCGACGGATTAACGCGATGACATTGTTGCGAGGTGGCCTGATAGCGGCAATTTTGTGTGCATTGCTCACGGTCTTATGCGCATGGGTACAATTGCCCATTCCGGCACTGGTGGCATTATTTTTCACCATCGCATTTTGTAGCGGCATCGGCACTGTTGGCGGGGCAGAGGCTATGAGTGCAGTAGGGACGCAGGAATCTGGAACGGCGTCTGCGTTGATGGGGATGAGCATGTTTGTCTTCGGCGGTATAGCCGCGCCATTGTCGGGAATTGGCGGAGAAACACTGTTAAAAATGAGTCTGGCAATTACGGTGTGTTATACGCTGGCATTGCTGGTTGCTCTCACCAGAATCGACAATCAAAAGTAA |

Note: The underlined sequences are designated as *eefR*, *eefA*, *eefB*, *eefC*, and *eefD*, respectively, with the shared region between *eefA* and *eefB* highlighted in red.

**Table S6** *Escherichia coli* isolates harboring the *eefRABCD* gene cluster on plasmids.

| **Accession** | **Description** | **Max Score** | **Total Score** | **Per. ident** | **Source** | **Phylogroup** |
| --- | --- | --- | --- | --- | --- | --- |
| CP165453.1 | *Escherichia coli* strain OXEC-515 plasmid unnamed, complete sequence | 13633 | 13633 | 99.51 | unknown | B2 |
| CP141085.1 | *Escherichia coli* strain KE48 plasmid unnamed1 | 13638 | 13638 | 99.52 | human | Unknown |
| CP134663.1 | *Escherichia coli* strain 64A:C plasmid unnamed1, complete sequence | 13295 | 13295 | 98.69 | animal | D |
| CP054253.1 | *Escherichia coli* strain STO_Bone4 plasmid pBone4_4 | 13620 | 13620 | 99.48 | human | Unknown |
| CP089259.1 | *Escherichia coli* strain D17EC0216 plasmid unnamed | 13206 | 13206 | 98.48 | human | Unknown |

**Figure S1** Purification and identification of EefR protein. A. SDS-PAGE analysis of the purified protein. M: Prestained Protein Marker (180 kDa, MP102-01); 1: Bacterial culture; 2: Cell lysate; 3: Supernatant; 4: Pellet; 5: Flow-through; 6: Wash buffer; 7: 60 mM imidazole; 8: 120 mM imidazole. B. Western-blotting analysis of EefR protein using anti-6His tag antibody. M: TrueColor Three-Color Prestained Protein Marker (10-180 kDa, GS1526); a: EefR protein.

**References**

1. Dai JS, Xu J, Shen HJ, et al. The induced and intrinsic resistance of *Escherichia coli* to sanguinarine is mediated by AcrB efflux pump. Microbiol Spectr. 2023;12(1):e03237-03223. doi: 10.1128/spectrum.03237-23
2. Jiang Y, Chen B, Duan C, et al. Multigene editing in the *Escherichia coli* genome via the CRISPR-Cas9 system. Appl Environ Microbiol. 2015;81(7):2506-2514. doi: 10.1128/AEM.04023-14
